# PET Imaging of CD8 via SMART for Monitoring the Immunotherapy Response

**DOI:** 10.1155/2021/6654262

**Published:** 2021-06-09

**Authors:** Lingyi Sun, Zhonghan Li, Yongyong Ma, Johannes Ludwig, Hyun S. Kim, Dexing Zeng

**Affiliations:** ^1^Center for Radiochemistry Research, Knight Cardiovascular Institute, Oregon Health & Science University, Portland 97229, USA; ^2^Department of Diagnostic and Interventional Radiology and Neuroradiology, University Hospital Essen, University of Duisburg-Essen, Essen 45147, Germany; ^3^Department of Radiology and Biomedical Imaging, Yale School of Medicine, New Haven 06510, USA; ^4^Department of Medicine, Yale Cancer Center, New Haven 06510, USA; ^5^Department of Diagnostic Radiology, Oregon Health & Science University, Portland 97229, USA

## Abstract

Imaging of CD8 receptors on T-cells by positron emission tomography (PET) has been considered a promising strategy for monitoring the treatment response to immunotherapy. In this study, a trial of imaging CD8 with our newly developed sequential multiple-agent receptor targeting (SMART) technology was conducted. Mice bearing a subcutaneous colorectal CT26 tumor received three times different immunotherapy treatments (PD1 or CTLA4 or combined). On either day 7 or day 14 after the first time treatment, the PET imaging study was performed with sequentially administered TCO-modified anti-CD8 antibody and ^64^Cu-labeled MeTz-NOTA-RGD. However, no positive response was detected, probably due to (1) inappropriate selection of biomarkers for the SMART strategy, (2) limited TCO modification on the anti-CD8 antibody, and (3) inadequate response of the CT26 tumor to the selected immunotherapies. Therefore, the potential of applying SMART in imaging CD8 was not demonstrated in this study, and further optimization will be necessary before it can be applied in imaging CD8.

## 1. Introduction

Immune checkpoint inhibitors have been widely used in the treatment of cancer [1, 2], and PET imaging of CD8 receptors on T-cells has been considered a promising strategy for monitoring patients' response to such immunotherapies [3, 4]. However, due to the slow body clearance of mAb, traditional immuno-PET imaging usually leads to sustained high radiodose uptake in blood and other normal organs, resulting in high background noise and raising the safety concern from the aspect of high radiodose exposure [5, 6]. To overcome this disadvantage, multiple pretargeting strategies have been proposed such as sequentially administrating one pair of TCO-modified mAb (TCO-mAb) and Tz-modified radioactive molecule (Tz-RM) [7, 8]. TCO and Tz are a pair of functional groups that ligate with each other very fast under ambient conditions [9, 10]. The administration of Tz-RM is usually performed several days after the administration of TCO-mAb to allow the sufficient accumulation of TCO-mAb at the tumor site and its concomitant clearance from normal organs, so that the radiodose could be trapped more in the tumor via the ligation between Tz-RM and TCO-mAb, decreasing the background noise. Furthermore, unlike the traditional approach in which the radioisotope is loaded on the intact mAb with a physical half-life time of several weeks [11], in the pretargeting strategy, the radioisotope is loaded on a small molecule possessing a much shorter half-life time (less than an hour) [12]. Therefore, those unconjugated Tz-RM will be cleared rapidly from the blood, significantly reducing the radiodose exposure to normal organs compared with the traditional approach using the intact labeled mAb. However, due to the lack of a tumor-targeting group, the tumor concentration of RM is low, leading to the insufficient tumor uptake caused by limited ligation between Tz-RM and previously administered TCO-mAb [8].

**Figure 1:**
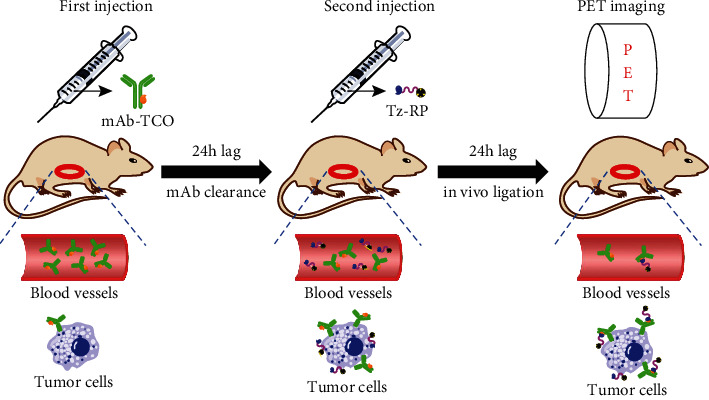
Workflow of SMART.

To address the low tumor uptake problem associated with the traditional pretargeting strategy, we developed the sequential multiple-agent receptor targeting (SMART) technology, featured by the sequential administration of a TCO-modified monoclonal antibody (TCO-mAb) and a Tz-modified radiolabeled peptide (Tz-RP) targeting two different biomarkers. As compared with Tz-RM in the traditional pretargeting strategy, the incorporation of a tumor-targeting peptide could increase the concentration of Tz-RP at the tumor site and prolong its tumor retention, leading to an improved tumor uptake. This technology synergized the advantages of both mAbs (long tumor retention) and peptides (tumor targeting and rapid nontumor clearance), rendering it a breakthrough strategy superior to those traditional mono-receptor-based strategies using only mAbs as the tumor-targeting vector (a comprehensive study regarding the development of SMART has been described in our other manuscript). The workflow of SMART technology (Figure 1) is illustrated as follows: (1) the preinjected TCO-mAb will accumulate at the tumor site, along with concomitant slow clearance from nontumor organs (in days); (2) the sequentially injected Tz-RP will also accumulate specifically at the tumor site (due to peptide-induced tumor targeting), along with concomitant rapid clearance from nontumor organs (in mins), which results in very low nontumor uptakes; and (3) over time, the Tz-RP will be continuously entrapped in the tumor due to *in situ* ligation with TCO-mAb, resulting in significantly increased tumor uptake.

In this proof-of-concept study, we conducted a trial of imaging CD8 after various immunotherapy treatments by SMART on mice bearing the subcutaneously implanted CT26 tumor. CT26 was a mouse colorectal cancer cell line [13], and immunotherapy treatments received by mice in this study included the PD1 treatment [14, 15], the CTLA4 treatment [14, 16], and their combination treatment [17]. The two biomarkers selected for the SMART in this study were CD8 and integrin alphaVbeta3. The selection of integrin alphaVbeta3 was because of its wide expression in various types of tumors [18, 19], and its existence in the CT26 tumor was also confirmed by an *ex vivo* biodistribution study (Figure S1).

## 2. Materials and Methods

### 2.1. Cell Line

The mouse colorectal cancer cell line, CT26, was purchased from the American Type Culture Collection (ATCC). Cells were cultured in RPMI 1640 supplemented with antibiotic-antimycotic solution (100 units/mL penicillin G, 250 ng/mL amphotericin B, and 100 units/mL streptomycin) and 10% fetal bovine serum (Invitrogen) at 37°C under 5% CO_2_.

### 2.2. Mouse Model

Four-week-old BALB/c mice were ordered from Taconic. All animal studies were conducted under a protocol approved by the Oregon Health & Science University Institutional Animal Care and Use Committee. Each mouse was subcutaneously implanted with 5∗10^5^ CT26 cells in PBS : Matrigel = 1 : 1 at the right flank. Tumors were allowed to grow for 10 days before receiving different treatments.

### 2.3. Immunotherapy Treatments

Mice bearing CT26 tumors were divided into four different treatment groups: (a) receiving 200 *μ*g anti-PD1 antibody on days 0, 3, and 5; (b) receiving 100 *μ*g anti-CTLA4 antibody on days 0, 3, and 5; (c) receiving 200 *μ*g anti-PD1 antibody+100 *μ*g anti-CTLA4 antibody on days 0, 3, and 5; and (d) receiving saline on days 0, 3, and 5.

### 2.4. Labeling of MeTz-NOTA-RGD with Radioisotopes

The ^64^Cu labeling of MeTz-NOTA-RGD was conducted by incubating 1 nmol MeTz-NOTA-RGD and 18.5 MBq ^64^Cu in 100 *μ*L 0.1 M NH_4_OAc buffer (pH~6. 8) at 70°C for 30 minutes. The labeling yield was above 95% based on HPLC monitoring.

### 2.5. PET Imaging Studies

PET imaging studies were conducted on day 7 and day 14 postinitial treatments. Mice bearing CT26 tumors were administrated with 100 *μ*g anti-CD8-TCO via the tail vein injection. The antibody was allowed to accumulate at the tumor site and clear from the blood for 24 h. Subsequently, 11.1 MBq MeTz-NOTA(^64^Cu)-RGD at a specific activity of 18.5 MBq/nmol was administered via the tail vein, and PET images were taken at 24 h postinjection. Mice were anesthetized with 2% isoflurane before small-animal PET/CT was performed. Static images were collected for 15 min. PET and CT images were coregistered with the Inveon Research Workstation (IRW) software (Siemens Medical Solutions). PET images were reconstructed with the ordered-subset expectation maximization 3-dimensional/maximum a posteriori probability algorithm, and the analysis of images was done using IRW.

### 2.6. Biodistribution Studies

Animals were euthanized after PET imaging. The blood, kidney, liver, lung, spleen, muscle, heart, bone, pancreas, intestine, and tumor were harvested. Radiation activities in tissue samples were measured in a gamma counter for 1 min each. Postweights were taken to determine the mass of tissue. Tissue weights and counts per minute (CPM) of ^64^Cu were used to calculate biodistribution.

## 3. Results and Discussion

Mice bearing the CT26 tumor were divided into 4 different treatment groups, including the PD1 treatment, CTLA4 treatment, PD1+CTLA4 combination treatment, and control IgG treatment. The SMART PET imaging study was conducted on day 7 and day 14 after the initial treatment. However, no positive response was detected.

**Figure 2:**
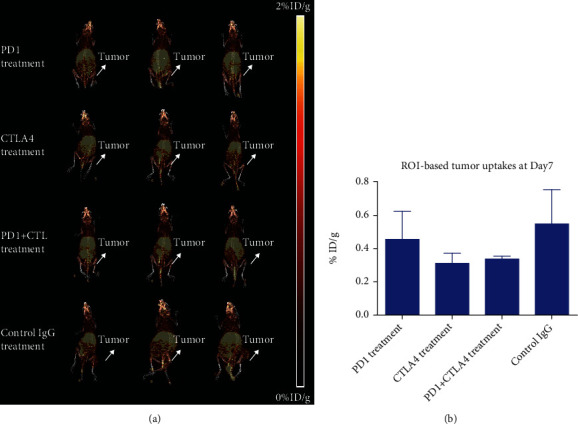
PET imaging study on day 7 after the initial treatment: (a) MIP spectrum; (b) tumor uptakes based on the region of interest analysis.

For the PET imaging study on day 7 (Figure 2(a) and Figure S2a), no significant difference in tumor uptakes was observed among all the treatment groups. Based on the region of interest analysis, tumor uptakes after the PD1 treatment, CTLA4 treatment, PD1+CTLA4 combination treatment, and control IgG treatment were found to be 0.455 ± 0.170%ID/g, 0.311 ± 0.062%ID/g, 0.338 ± 0.017%ID/g, and 0.548 ± 0.208%ID/g, respectively (Figure 2(b)).

**Figure 3:**
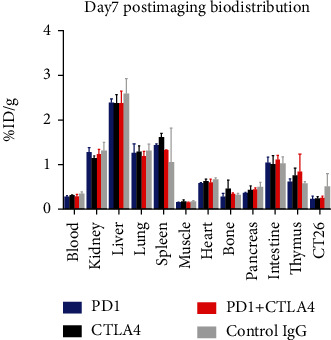
Day 7 postimaging biodistribution study.

The postimaging biodistribution study revealed that the major uptakes were by the kidney, liver, lung, spleen, and intestine (Figure 3). Specifically, kidney uptakes were found to be ranging from 1.120%ID/g to 1.290%ID/g, liver uptakes were found to be ranging from 2.354%ID/g to 2.569%ID/g, lung uptakes were found to be ranging from 1.161%ID/g to 1.296%ID/g, spleen uptakes were found to be ranging from 1.035%ID/g to 1.594 ± 0.084%ID/g, and intestine uptakes were found to be ranging from 0.990%ID/g to 1.092 ± 0.092%ID/g. In the other organs, uptake values were much less than 1%ID/g (Table 1). In particular, tumor uptakes were found to be ranging from 0.209%ID/g to 0.486%ID/g. Although these values were a bit different from those obtained via the ROI analysis (ranging from 0.311%ID/g to 0.548%ID/g), conclusions drawn from these two sets of data were consistent that no significant difference was detected among the four different treatment groups.

**Figure 4:**
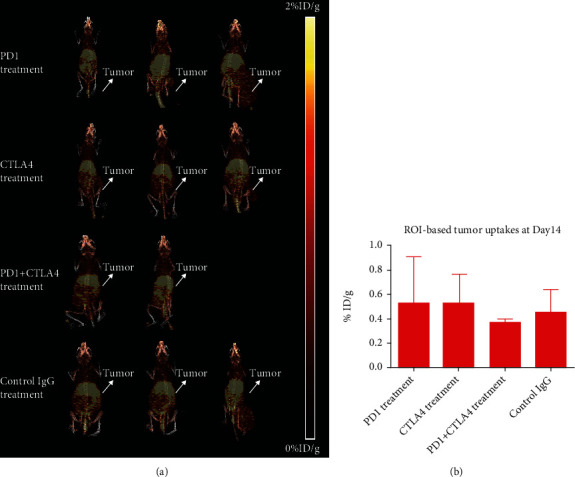
PET imaging study on day 14 after the initial treatment: (a) MIP spectrum; (b) tumor uptakes based on the region of interest analysis.

For the PET imaging study on day 14 (Figure 4(a) and Figure S2b), similar results were obtained. No significant difference in tumor uptakes was observed among all treatment groups. Based on the region of interest analysis, tumor uptakes after the PD1 treatment, CTLA4 treatment, PD1+CTLA4 combination treatment, and control IgG treatment were found to be 0.529 ± 0.382%ID/g, 0.528 ± 0.234%ID/g, 0.369 ± 0.029%ID/g, and 0.457 ± 0.183%ID/g (Figure 4(b)).

**Figure 5:**
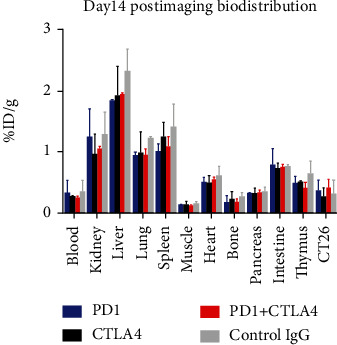
Day 14 postimaging biodistribution study.

The postimaging biodistribution study again revealed that the major uptakes were by the kidney, liver, lung, spleen, and intestine (Figure 5). Specifically, kidney uptakes were found to be ranging from 0.955%ID/g to 1.291%ID/g, liver uptakes were found to be ranging from 1.842%ID/g to 2.332%ID/g, lung uptakes were found to be ranging from 0.938%ID/g to 1.220%ID/g, spleen uptakes were found to be ranging from 1.003 to 1.411%ID/g, and intestine uptakes were found to be ranging from 0.731 to 0.769%ID/g. In the other organs, uptake values were much less than 1%ID/g (Table 2).

Based on PET imaging results, no positive response was detected for all the three different treatment groups. Uptakes found in the kidney and intestine could be due to the body excretion of metabolites of hot tracers, while those found in the liver, spleen, and thymus may indicate nonspecific (for the liver) or specific uptakes (for the spleen and thymus) of anti-CD8-TCO conjugated by the MeTz-NOTA-RGD in the blood.

Reasons for the failure of detecting positive response could be due to the following. (1) The distribution pattern of the two selected biomarkers is not ideal for the SMART strategy. Since tissue concentrations of chemical tools will be much lower than their initial blood concentrations, the proximity of the two chosen biomarkers could be essential for the ligation efficiency. However, CD8 receptors are distributed on the T-cells while the integrin alphaVbeta3 receptors are distributed on either cancer cells or endothelial cells. Therefore, it will be difficult for chemical tools targeting different types of cells to ligate with each other. (2) The average number of TCO on each CD8 antibody was not sufficient (2 TCO/mAb), which limited the overall amount of TCO available, thus affecting the efficacy of the *in vivo* ligation. (3) The response of the CT26 tumor to the selected immunotherapy was poor. Based on the reference, the positive response ratios of CT26 tumors towards different treatments were 25%, 50%, and 75% for the PD1 treatment, CTLA4 treatment, and PD1+CTLA4 treatment, respectively [17]. Therefore, at least for the PD1 treatment, the sample size will be too small to ensure the existence of the positive case.

## 4. Conclusions

In this study, a trial of imaging CD8-positive T-cells for monitoring the response to different immunotherapy treatments based on the SMART technology was conducted. However, no positive response was detected in this PET imaging study. The problem may be resolved with a proper selection of one pair of biomarkers, an improved modification of the antibody with a higher number of TCO, and a larger sample size.

## Figures and Tables

**Table 1:** Postimaging biodistribution data for day 7 (values are %ID/g ± SD).

Organ	PD1	CTLA4	PD1+CTLA4	Control IgG
Blood	0.256 ± 0.019	0.291 ± 0.009	0.266 ± 0.04	0.328 ± 0.033
Kidney	1.255 ± 0.104	1.120 ± 0.05	1.213 ± 0.102	1.290 ± 0.184
Liver	2.368 ± 0.083	2.354 ± 0.197	2.362 ± 0.264	2.569 ± 0.338
Lung	1.247 ± 0.194	1.266 ± 0.133	1.161 ± 0.106	1.296 ± 0.139
Spleen	1.411 ± 0.028	1.594 ± 0.084	1.301 ± 0.011	1.035 ± 0.761
Muscle	0.131 ± 0.006	0.153 ± 0.03	0.127 ± 0.009	0.153 ± 0.02
Heart	0.566 ± 0.006	0.604 ± 0.046	0.578 ± 0.069	0.649 ± 0.03
Bone	0.225 ± 0.072	0.441 ± 0.188	0.314 ± 0.032	0.297 ± 0.034
Pancreas	0.345 ± 0.011	0.410 ± 0.087	0.419 ± 0.031	0.475 ± 0.104
Intestine	1.020 ± 0.128	0.990 ± 0.189	1.092 ± 0.092	1.002 ± 0.149
Thymus	0.594 ± 0.065	0.732 ± 0.159	0.821 ± 0.392	0.556 ± 0.038
CT26	0.209 ± 0.067	0.223 ± 0.033	0.223 ± 0.045	0.486 ± 0.291

**Table 2:** Postimaging biodistribution data for day 14 (values are %ID/g ± SD).

Organ	PD1	CTLA4	PD1+CTLA4	Control IgG
Blood	0.323 ± 0.195	0.226 ± 0.061	0.234 ± 0.022	0.351 ± 0.170
Kidney	1.252 ± 0.451	0.955 ± 0.320	1.052 ± 0.040	1.291 ± 0.350
Liver	1.842 ± 0.019	1.923 ± 0.473	1.882 ± 0.084	2.332 ± 0.362
Lung	0.938 ± 0.054	0.981 ± 0.339	0.939 ± 0.089	1.220 ± 0.029
Spleen	1.003 ± 0.113	1.242 ± 0.222	1.086 ± 0.157	1.411 ± 0.38
Muscle	0.122 ± 0.018	0.122 ± 0.05	0.110 ± 0.01	0.142 ± 0.035
Heart	0.499 ± 0.071	0.472 ± 0.117	0.536 ± 0.033	0.611 ± 0.143
Bone	0.158 ± 0.109	0.227 ± 0.114	0.166 ± 0.056	0.259 ± 0.061
Pancreas	0.313 ± 0.022	0.318 ± 0.072	0.316 ± 0.039	0.348 ± 0.066
Intestine	0.769 ± 0.273	0.731 ± 0.075	0.754 ± 0.033	0.761 ± 0.017
Thymus	0.48 ± 0.102	0.509 ± 0.006	0.395 ± 0.105	0.644 ± 0.189
CT26	0.353 ± 0.174	0.264 ± 0.136	0.407 ± 0.127	0.303 ± 0.216

## Data Availability

The data in this study are available from corresponding authors on reasonable request.

## Supplementary Materials

Supplementary Materials Supplementary Figure S1: ex vivo biodistribution study of RGD on the subcutaneous CT26 mouse model. Supplementary Figure S2: axial PET images: (a) day 7; (b) day 14.
