# Supplementary material for: PET Imaging of CD8 via SMART for Monitoring the Immunotherapy Response
Source: Biomed Res Int. 2021 Jun 9;2021:6654262. doi: 10.1155/2021/6654262 (PMC8211506; doi:10.1155/2021/6654262)
Supplement: Supplementary Materials — Supplementary Figure S1: ex vivo biodistribution study of RGD on the subcutaneous CT26 mouse model. Supplementary Figure S2: axial PET images: (a) day 7; (b) day 14. [file 6654262.f1.docx]

**Supporting information**

1. **Materials and equipment**

Aqueous solutions were prepared using ultrapure water (resistivity, 18 MΩ*cm). ^64^Cu was obtained from Washington University (St. Louis, MO). The PD-1, CTLA4 and anti-CD8 antibodies were purchased from Bio X cell (Lebanon, NH). RGD was purchased from Peptides International (Louisville, Kentucky). All other chemicals were purchased from Sigma-Aldrich Chemical Co. (St. Louis, MO) or Fisher Scientific (Pittsburgh, PA), unless otherwise specified. ESI-MS were measured on a Waters LCT-Premier XE LC-MS station (Milford, MA). Luna C-18 HPLC columns were purchased from Phenomenex (Torrance, CA, USA). SUPEROSE 12 10/300 GL size exclusion columns for FPLC were purchased from Fisher Scientific (Pittsburgh, PA). Both HPLC and FPLC were performed on a Waters 1525 Binary HPLC pump (Milford, MA) with a Waters 2489 UV/visible detector and a model 106 Bioscan radioactivity detector for the purification of peptide conjugates and analysis of their radiolabeled conjugates using two elution buffers (0.1 v% TFA in de-ionized water as elution buffer A and 0.1 v% TFA in acetonitrile as elution buffer B). PET/CT data were acquired using an Inveon Preclinical Imaging Station (Siemens Medical Solutions).

1. **Preparation of antiCD8-TCO**

To 1ml 2mg/ml antiCD8 solution was added saturated NaHCO_3_ for adjusting the pH to 8.5. 0.27µmole (20equiv) TCO-PEG4-NHS dissolved in 50µl DMSO was subsequently added, and the reaction mixture was gently stirred at 4℃ for overnight. Excess TCO-PEG4-NHS was subsequently removed by dialysis or desalting columns to give antiCD8-TCO.

The average number of TCO on each antibody was quantified by tetrazine-Cy5. In particular, to 0.1nmole prepared antiCD8-TCO was treated with 2nmole tetrazine-Cy5. The reaction mixture was stirred at room temperature for 1h and then monitored by FPLC at the wavelength of 280nm and 646nm. The peak area of antiCD8-TCO at 646nm was divided by the peak area of the same amount of antiCD8-TCO at 280nm, and the resulting value was further normalized by extinction coefficients of Cy5 at 646nm (250000M^-1^cm^-1^) and IgG at 280nm (210000M^-1^cm^-1^) to give the average number of TCO per mAb.

1. **Preparation of MeTz-PEG-NOTA-PEG-RGD**

To 1mmole N_3_-NOTA(tBu)-COOH dissolved in 5ml DMF was added 1.5mmole HATU, 5mmole TEA, and 2mmole NH_2_-PEG_3_-NH(Boc). The reaction mixture was stirred at room temperature for overnight and then partitioned between 25ml DCM and 25ml H_2_O. The DCM layer was separated and washed by 50ml water for three times. DCM was then removed, and the residue was purified by column chromatography to 0.6mmole give N_3_-NOTA(tBu)-PEG_3_-NH(Boc) **1**. Yield 60%.

To 10µmole RGD in 0.5ml DMF was added 200µmole DIEA and 20µmole BCN-PEG_4_-NHS. The reaction mixture was stirred at room temperature for 1h. DMF was removed by the lyophilizer. The residue was dissolved in 25% acetonitrile in water and purified by semi-preparative HPLC (with a gradient changing from 0% acetonitrile with 0.1v% TFA to 50% acetonitrile with 0.1v% TFA within 20min) to obtain 7.1µmole RGD-PEG_4_-BCN **2**. Yield 71%.

To 10µmole N_3_-NOTA(tBu)-PEG_3_-NH(Boc) **1** in 0.4ml DMF:H2O = 3:1 was added 5µmole RGD-PEG_4_-BCN **2**. The reaction mixture was stirred at room temperature for overnight. Solvent was removed by the lyophilizer. The residue was dissolved in 25% acetonitrile in water and purified by semi-preparative HPLC (with a gradient changing from 0% acetonitrile with 0.1v% TFA to 60% acetonitrile with 0.1v% TFA within 20min) to obtain 2.9µmole RGD-PEG_4_-NOTA(tBu)-PEG_3_-NH(Boc) **3**. Yield 58%.

2µmole RGD-PEG_4_-NOTA(tBu)-PEG_3_-NH(Boc) **3** was dissolved in 0.5ml 95%TFA, and the reaction mixture was stirred at room temperature for 2h. TFA was then completely removed. The residue was lyophilized to give RGD-PEG_4_-NOTA-PEG_3_-NH_2_ **4**, which was directly used in the next step without further purification.

RGD-PEG_4_-NOTA-PEG_3_-NH_2_ **4** was re-dissolved in 0.1ml DMF. 20µmole DIEA and 5µmole methyltetrazine-NHS were subsequently added. The reaction mixture was stirred at room temperature for 1h. DMF was removed by the lyophilizer. The residue was dissolved in 25% acetonitrile in water and purified by semi-preparative HPLC (with a gradient changing from 0% acetonitrile with 0.1v% TFA to 60% acetonitrile with 0.1v% TFA within 20min) to obtain 1.2µmole MeTz-PEG-NOTA-PEG-RGD **5**. Yield 60%. ESI-MS, m/z (M+2H)^2+^/2: Calcd 901.4658; found 901.4714.


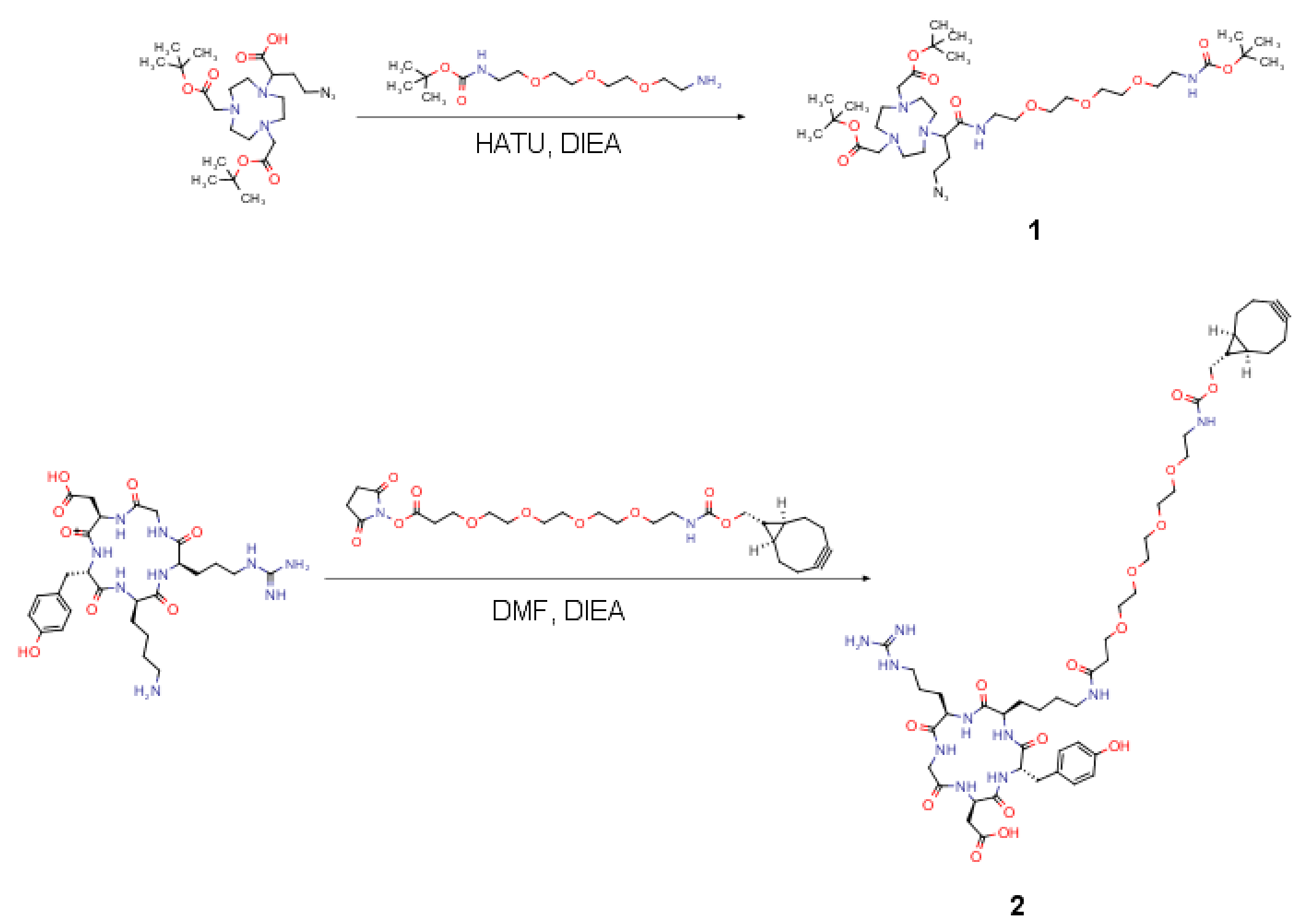


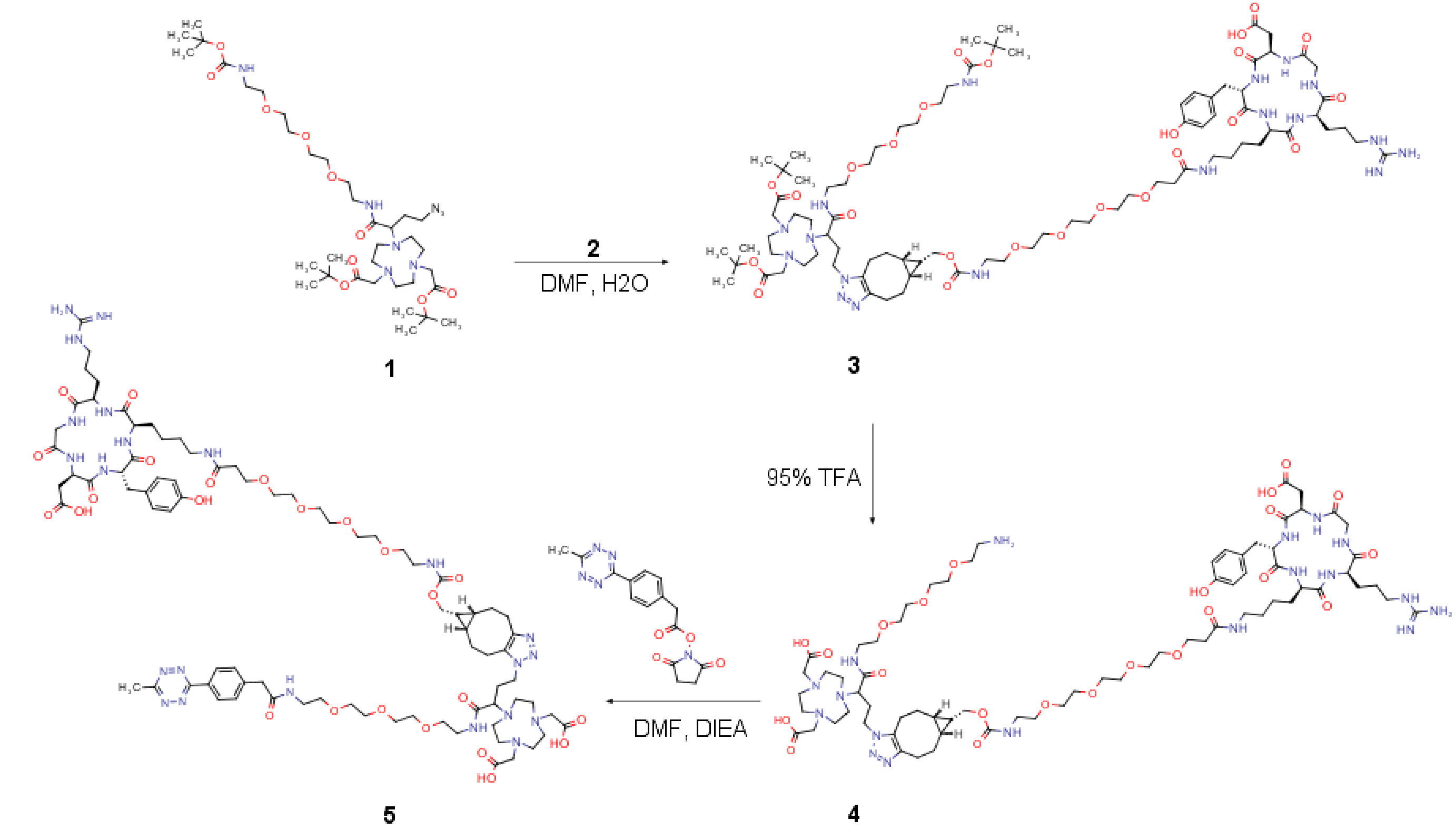


**Scheme S1.** Synthesis of MeTz-PEG-NOTA-PEG-RGD.

1. **Biodistribution for confirming the existence of integrin alphaV beta 3 in the CT26 tumor**

Since the major feature of SMART as compared with the traditional pre-targeting is that its second reagent bearing a tumor targeting moiety, it is of importance to confirm the availability of the corresponding receptor at the tumor site before applying SMART. Therefore, we first confirmed the existence of integrin alphaV beta 3 in the CT26 tumor by conducting an *ex vivo* biodistribution study of ^64^Cu-RGD-NODAGA (Herein, RGD is the abbreviation of the peptide c(RGDyK).). Results demonstrated that ^64^Cu-RGD-NODAGA (0.1nmole per mouse with a specific activity of 18.5MBq/nmole) exhibited an obvious tumor uptake of 1.5±0.5%ID/g, indicating that integrin alphaV beta 3 is expressed in the CT26 and MeTz-PEG-NOTA-PEG-RGD can be applied in the subsequent SMART imaging study.


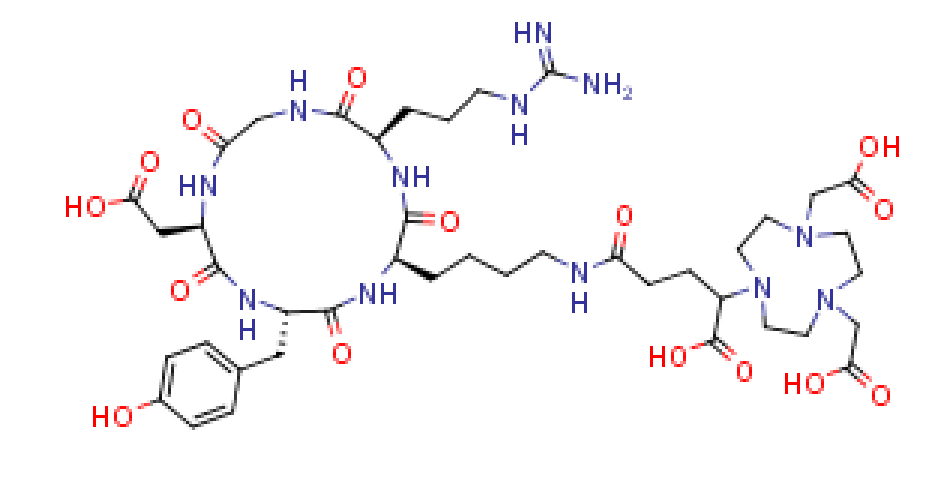


**(a)**


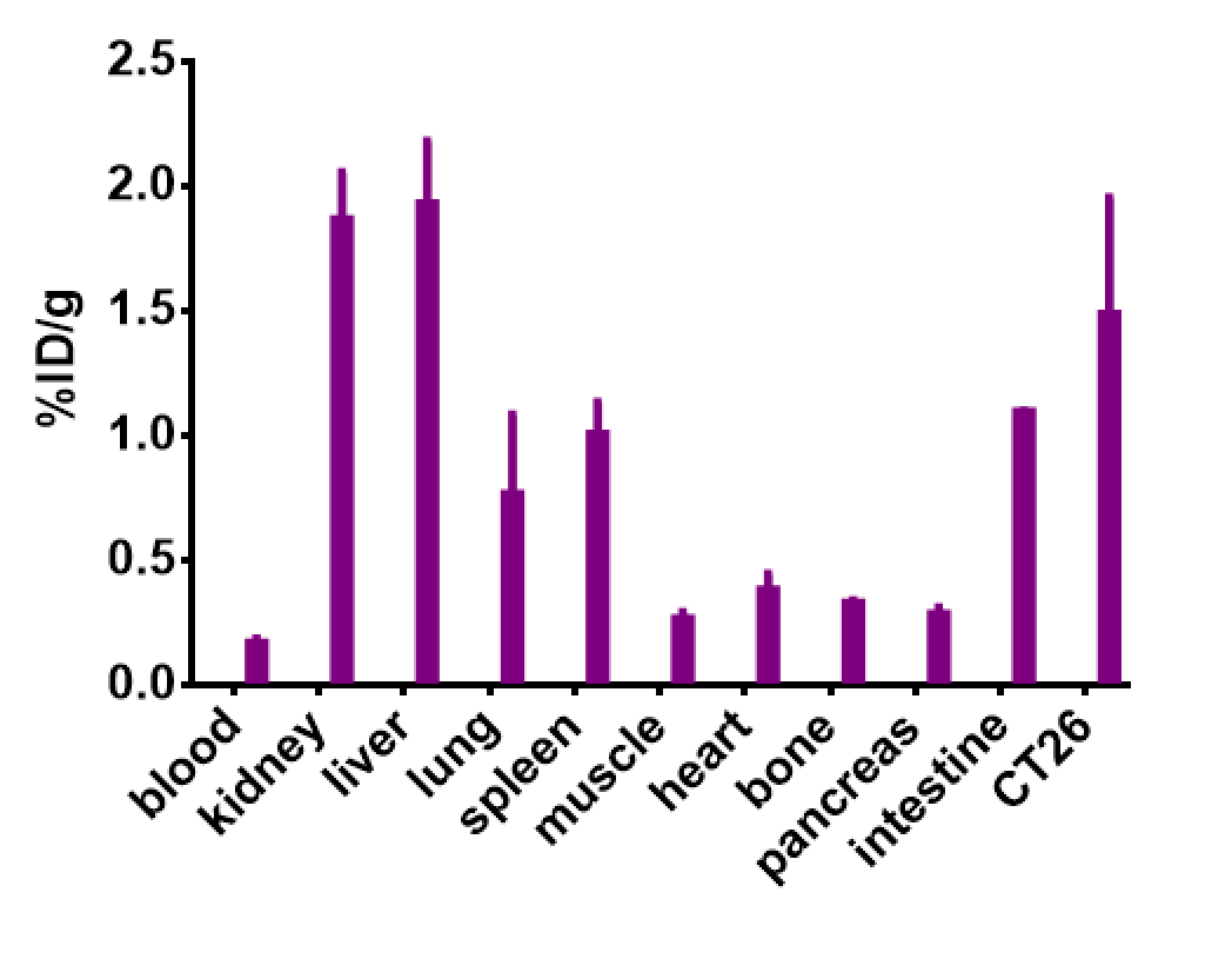


**(b)**

**Figure S1: (a)** Structure of RGD-NODAGA; **(b)** *Ex-vivo* 1h post injection biodistribution study of RGD-NODAGA on the subcutaneous CT26 mouse model.

1. **Axial PET images**


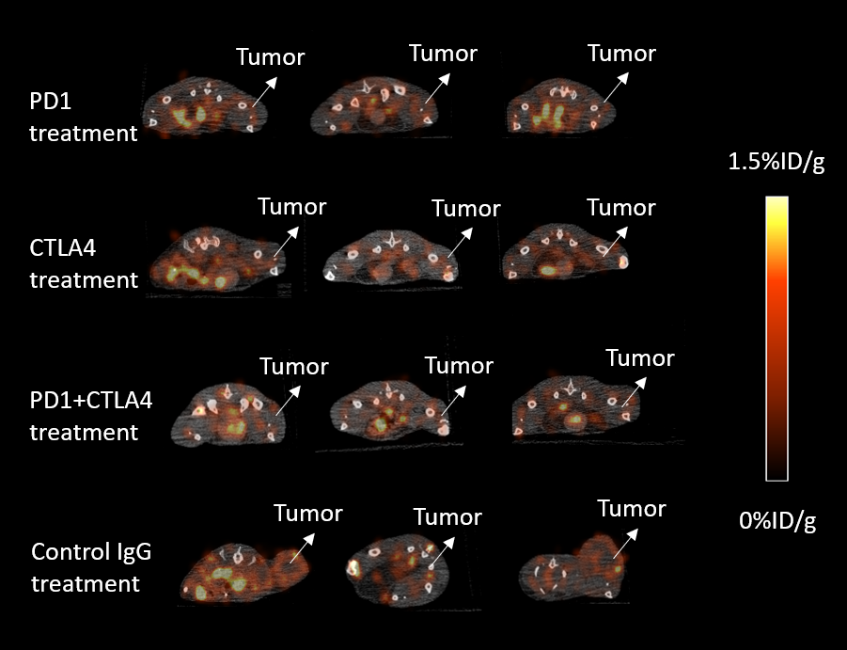

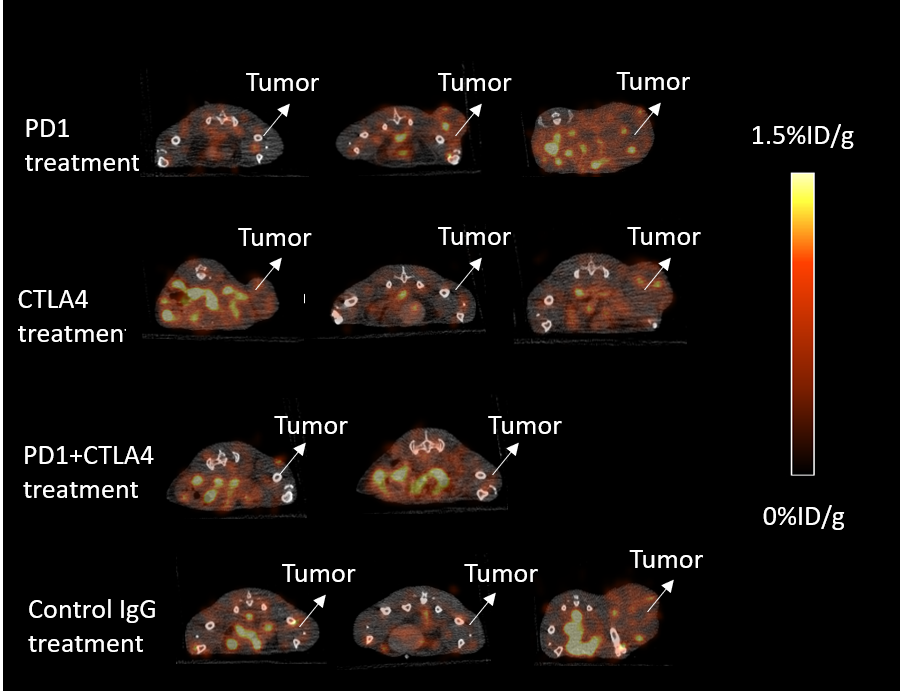


**(a) (b)**

**Figure S2:** Axial PET images: (a) Day7; (b) Day14.
